# Intravenous paracetamol and dipyrone for postoperative analgesia after day-case tonsillectomy in children: a prospective, randomized, double blind, placebo controlled study

**DOI:** 10.5935/1808-8694.20130015

**Published:** 2015-10-14

**Authors:** Aysu Inan Kocum, Mesut Sener, Esra Caliskan, Nesrin Bozdogan, Deniz Micozkadioglu, Ismail Yilmaz, Anis Aribogan

**Affiliations:** Assist. Prof. (Baskent University Department of Anesthesiology and Reanimaton); Assist. Prof. (Baskent University Department of Otorhinolaryngology and ENT surgery); Prof. (Baskent University Department of Anesthesiology and Reanimaton). Baskent University Faculty of Medicine Adana Teaching and Research Center Yuregir-ADANA /TURKEY

**Keywords:** analgesics, opioid, pain clinics, pediatrics, tonsillectomy

## Abstract

Tonsillectomy is associated with severe postoperative pain for which, several drugs are employed for management.

**Objective:**

In this double-blind, placebo-controlled study we aimed to evaluate the efficacy of intravenous paracetamol and dipyrone when used for post-tonsillectomy analgesia in children.

**Method:**

120 children aged 3-6 yr, undergoing tonsillectomy with or without adenoidectomy and/ or ventilation tube insertion were randomized to receive intraoperative infusions of paracetamol (15 mg/kg), dipyrone (15 mg/kg) or placebo (0.9% NaCl). Evaluation was carried out at 0.25, 0.50, 1, 2, 4, 6h postoperatively. Pethidine 0.25 mg/kg was utilized as rescue analgesic. Cumulative pethidine requirement was the primary outcome. Pain intensity measurement, pain relief, sedation level, nausea and vomiting, postoperative bleeding and any other adverse effects were noted.

**Results:**

No significant difference was found in pethidine requirement between paracetamol and dipyrone groups. Cumulative pethidine requirement was significantly less in paracetamol and dipyrone groups *vs.* placebo. No significant difference was observed between groups in postoperative pain intensity scores throughout the study.

**Conclusion:**

Intravenous paracetamol is found to have a similar analgesic efficacy as intravenous dipyrone and they both help to reduce the opioid requirement for postoperative analgesia in pediatric day-case tonsillectomy.

## INTRODUCTION

Tonsillectomy with or without adenoidectomy is associated with severe postoperative pain and presently forms a major part of day-case pediatric anesthetic mana-gement^1^. It is important to provide effective and safe pain control in this patient group allowing early ambulation. Potential for respiratory depression, nausea and vomiting limits the use of opioid analgesics particularly in this patient group when care of the patient is parents' responsibility after early discharge^2^^,^^3^ particularly following a surgical procedure involving the upper respiratory pathway^4^. Dipyrone has a substantial use in many centers^5^, ^6^, ^7^, ^8^ however it is reluctantly used and even taken off the market in some countries^9^ due to data that it may increase the risk of agranulocytosi^10^. Intravenous paracetamol with a well established safety profile may be a reasonable alternative in these patients^11^. Paracetamol in oral and rectal suppository forms have long been used for postoperative analgesia in children^12^; however irregular bioavailability of rectal form and temporary prohibition of oral intake in post tonsillectomy patients are important factors limiting the use of paracetamol in immediate postoperative management of pain following tonsillectomy^13^.

Recently, following introduction of intravenous form of paracetamol with more predictable pharmacodynamic properties when compared with its other forms^11^, safety and efficacy of intravenous paracetamol has been tested in various postoperative clinical scenarios^14^ but there are still only a few publications about efficacy of intravenous paracetamol administration for postoperative pain management in pediatric age group^2^^,^^14^, ^15^, ^16^, ^17^, ^18^.

Particularly there is lack of data involving intravenous paracetamol and intravenous dipyrone in a placebo controlled setting which may be used as evidence that intravenous paracetamol might be a reasonable alternative for dipyrone for post-tonsillectomy pain management among pediatric age group. The aim of this study is to evaluate the efficacy of intravenous paracetamol and intravenous dipyrone in a placebo controlled setting for postoperative pain management during day case tonsillectomy in pediatric age group.

## METHOD

The protocol was approved by the Ethics Committee of University Faculty of Medicine (project no: KA08/46). Following written informed parental consent and verbal child assent had been obtained, consecutive patients age between 3-6 year who were scheduled for elective day case tonsillectomy together with or without adenoidectomy and/ or ventilation tube insertion in our university hospital were screened for eligibility for enrollment in this prospective, randomized, double blind, placebo controlled study.

Extreme care is undertaken in order to manage pain in all groups including the placebo group by strict adherence for rescue opioid analgesic medication in each and every assessment interval. Inclusion criteria were patient physical status ASA I; scheduled day-case elective operation. Exclusion criteria were emergency surgery; known hypersensitivity to the drugs under study; history of renal, hepatic, respiratory or cardiac disease; neurological or neuromuscular disorders; known glucose 6 phosphate dehydrogenase deficiency concomitant medication (anti-convulsants, corticosteroids, period before the study.

Following screening, eligible patients were randomized to receive a single dose of either intravenous paracetamol 15 mg/kg premixed with 0.9% NaCl to a total of 50 ml, intravenous dipyrone 15 mg/kg premixed with 0.9% NaCl to a total of 50 ml or 50 ml of 0.9% NaCl as placebo according to a pre-generated randomization scheme created by the web site Randomization.com (http://www.randomization.com). Patients, all care givers and the clinical observers who scored were blinded to the allocated treatment of the individual patient. All study medications were prepared by a clinician unaware of the patient's allocated study group in identical infusion pumps. Infusions were administered by a blinded attending physician. All study medications were infused as a single dose following induction of general anesthesia. All patients were preme-dicated with 0.3 mg/kg oral midazolam and 0.2 mg/kg intravenous dexamethasone before surgery.

General anesthesia was induced by thiopental 3-5 mg/kg, fentanyl 1 mg/kg and vecuronium 0.1 mg/ kg. After tracheal intubation, mechanical ventilation was initiated, and a 50% mixture of N_2_/O_2_ was administered throughout the surgery. Anesthesia was maintained with isoflurane 1.0-1.2 MAC. No additional opioid was given intraoperatively. At the end of the operation residual neuromuscular block was reversed with neostigmine 0.04 mg/kg and atropine 0.02 mg/kg. Endotracheal tube was removed when respiration was regular and adequate according to the discretion of the attending physician. Patients were transferred to post anesthesia care unit after the removal of endotracheal tube. Investigators recorded the study variables 0.25, 0.5, 1, 2, 4, and 6h after arrival to post anesthesia care unit. Pain intensity was assessed by Children's Hospital East Ontario Pain Scale (CHEOPS) with range of scores 4-13^19^.

Pain relief (PR) was assessed by a 5 point verbal scale (16) rated by the investigator indicating 0 = none, 1 = a little, 2 = moderate, 3 = a lot, 4 = complete. Sedation level was assessed by a 4 point scale rated by the investigator indicating 0 = eyes open spontaneously, 1 = eyes open to speech, 2 = eyes open to gentle shaking, 3 = unarousable. Nausea and vomiting were assessed by a 3 point scale rated by the investigator indicating 0 = no nausea, 1 = nausea without vomiting in the evaluation period, 2 = vomiting in the evaluation period. In case of CHEOPS score > 6 and/ or PR score < 2, the patient received 0.25 mg/kg pethidine in to a maximum total dose of 1.5 mg/kg in 6 hours as rescue analgesic medication until CHEOPS score was ≤ 6 and PR ≥ 2. Metoclopramide 0.1 mg/kg was used as an antiemetic for recurrent nausea or vomiting. Duration of post anesthesia care unit stay was recorded. Any hemorrhage requiring intervention at the surgical site and other adverse effects attributable to any drug effect were also noted. Patients meeting standard discharge characteristics (awake, hemodynamically stable, maintaining a patent airway without support, protective reflexes intact, free of adenotonsillary persistent bleeding, and comfortable) were discharged from post anesthesia care unit.

A priori power analysis was performed on the basis of the cumulative pethidine requirement data obtained from the first 15 patients in the placebo group among the present study. The results of the first 15 patients in the placebo group showed that mean cumulative pethidine requirement was 0.62 ± 0.29 mg/kg during the first 6 hours postoperatively. In order to detect a 30% reduction in the dose of cumulative pethidine requirement between an active treatment group and placebo with 80% power at a = 0.05; power analysis suggested a sample size of 38 per each group. Analysis was performed by Power and Precision^TM^ (Biostat Inc., Englewood, NJ) statistical program. Differences among the three groups were analyzed by an analysis of variance test or its nonparametric counterpart, the Kruskal-Wallis test. The homogeneity of variances was calculated with the Levene test and the Lilliefors significance correction test. Post hoc analyses were performed with the Bonferroni test. Either the chi-square or Fisher's exact test was used to analyze categorical variables when appropriate. Differences were considered statistically significant at *p <* 0.05. Data were expressed on means ± SD or median (25-75). Statistical calculations were performed with SPSS software (Statistical Package for the Social Sciences, version 11.0; SSPS Inc, Chicago, IL).

## RESULTS

**Figure 1:**
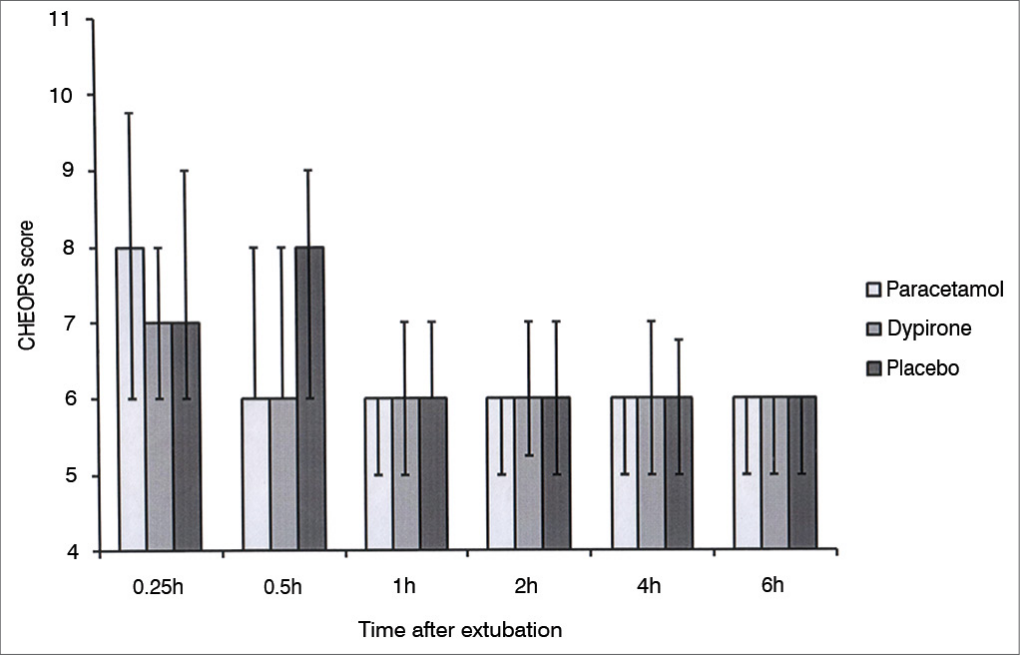
CHEOPS score among groups. Data expressed as Median (25-75 centile). No significant difference at any time interval between any group.

One hundred thirty eight patients were screened to participate in this study. During screening nine patients were found to be not eligible for the study, seven patients' parents declined to give consent and two patients were excluded due to other reasons. A total of 120 patients constituted the study population. Each group comprised of 40 cases. There were no differences among groups with regard to age distribution, weight, sex, type of the operation, and duration of the operation (Table 1). There was no difference in the CHEOPS score between groups at any time interval during follow up (Figure 1).

**Table 1:** Demographic data and operative characteristics.

	Paracetamol (n = 40)	Dipyrone (n = 40)	Placebo (n = 40)	*p*
Age (yr)	4.7 ± 1.0	4.6 ± 0.9	4.3 ± 1.0	0.222
Sex (male/female)	29/11	27/13	19/21	0.05
Weight (kg)	16.9 ± 3.3	18.0 ± 5.2	17.7 ± 4.5	0.53
Type of the operation				0.901
Tonsillectomy	34	35	33	
Adenotonsillectomy	4	4	4	
Adenotonsillectomy + ETI	2	1	3	
Duration of surgery (min)	36.9 ± 12.1	42.1 ± 12.7	36.6 ± 14.6	0.123

Data expressed as mean ± standard deviation. ETI: ear tube insertion.

**Figure 2:**
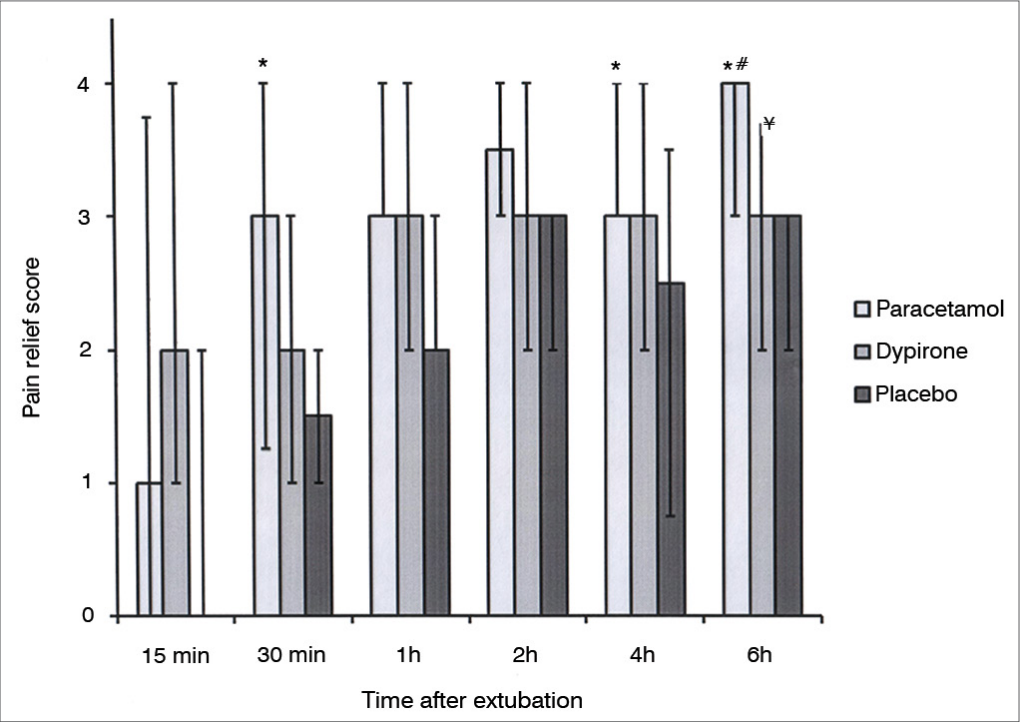
Pain relief score among groups. Data expressed as Median (25-75 centile). * Pain relief score significantly higher in iv.paracetamol group *vs.* placebo in 0.5,4 and 6 h. (*p*: 0.04; *p*: 0.01; *p* less than 0.001 respectively). ^#^ Pain relief score significantly higher in iv. paracetamol group *vs.* iv. dipyrone in 6h. (*p*: 0.04). ^¥^ Pain relief score significantly higher in iv dipyrone *vs.* placebo in 6h. (*p*: 0.03).

There was no difference between groups among pain relief score at 0.25, 1, and 2h during follow up; however pain relief score in paracetamol group was significantly higher than placebo group at 0.5 and 4h follow up (*p* = 0.04; *p* = 0.01 respectively). There were no differences between dipyrone *vs*. placebo and dipyrone *vs.* paracetamol groups with regard to pain relief score at 0.5h follow up. At 6h follow up, pain relief score was significantly higher in paracetamol group when compared with placebo (*p*< 0.001) and dipyrone (*p* = 0.04) groups. Also at 6h follow up pain relief score was significantly higher in dipyrone group than placebo group (*p* = 0.03) (Figure 2).

**Figure 3:**
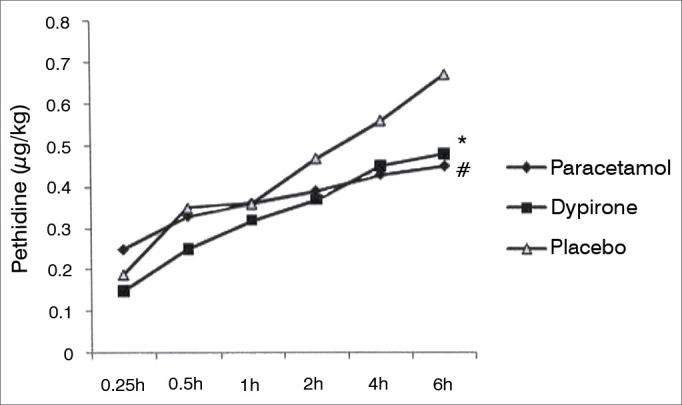
Cumulative pethidine requirement. Data expressed as mean ± SD. * Dipyrone significantly decrease pethidine requirement compared to placebo (*p*: 0.03). ^#^ Paracetamol significantly decrease pethidine requirement compared to placebo (*p*: 0.01).

**Figure 4:**
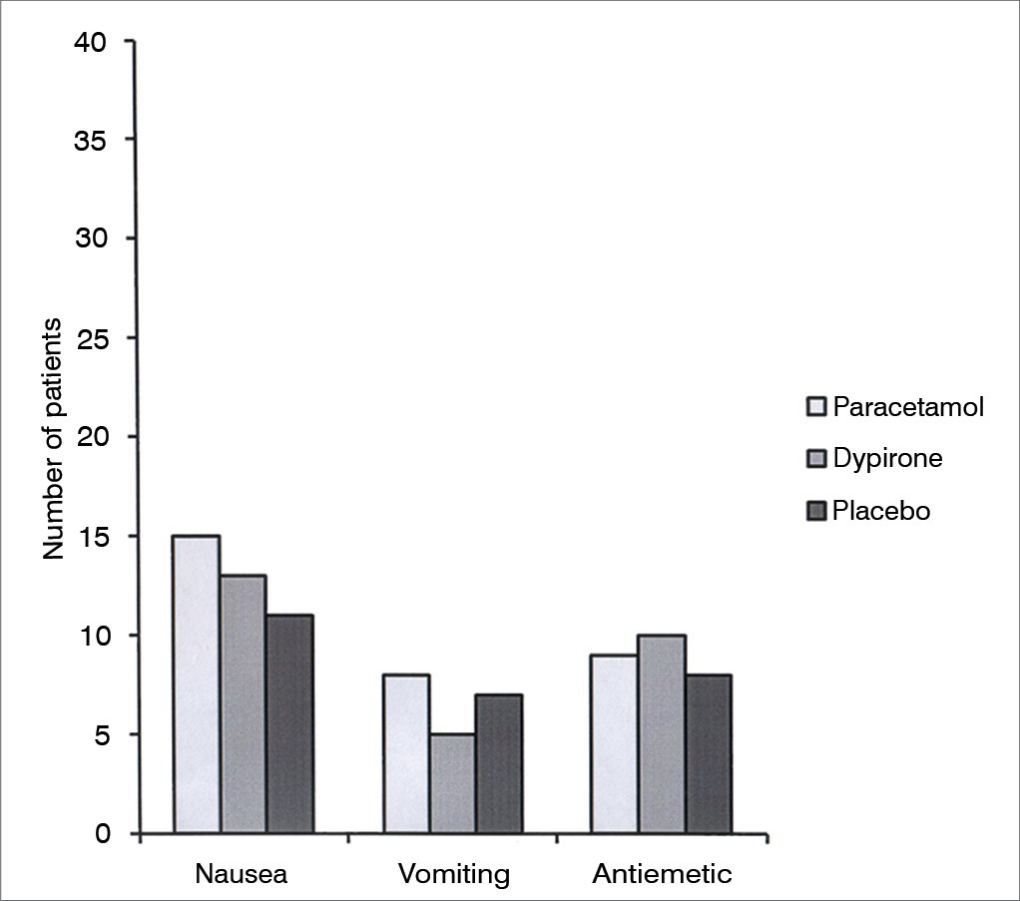
Data among nausea and vomiting.

**Figure 5:**
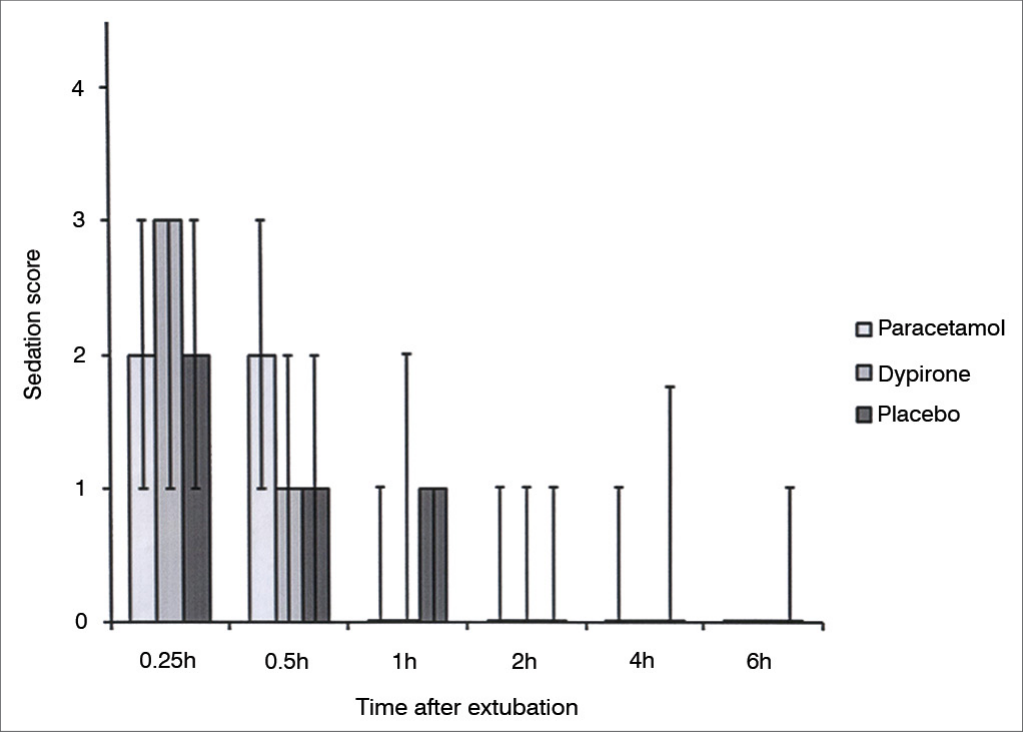
Sedation score among groups. Data expressed as Median (25-75 centile) No significant difference at any time interval between any group.

Cumulative pethidine use as a rescue analgesic was not different among groups at 0.25, 0.5, 1, 2, and 4h follow up; however at 6h follow up both paracetamol *vs.* placebo and dipyrone *vs*. placebo comparisons revealed significantly lower rescue analgesic requirement in active treatment groups (0.45 ± 0.30 mg/kg [95% CI 0.35-0.55] vs 0.67 ± 0.38 mg/kg [95% CI 0.55-0.79], *p* = 0.01; 0.48 ± 0.30 mg/kg [95% CI 0.38-0.57], *vs*. 0.67 ± 0.38 mg/kg [95% CI 0.55-0.79], *p* = 0.03 respectively). Cumulative pethidine requirement was similar between paracetamol and dipyrone groups. (Figure 3). Number of patients who had nausea, vomiting or antiemetic administration during the study were similar among all three groups (Figure 4). There was no difference among sedation score between groups at any time interval during follow up (Figure 5).

Duration of stay in the post anesthesia care unit was similar between groups (paracetamol 36.12 ± 14.52 min (95% CI 31.48-40.76); dipyrone 41.02 ± 14.28 min (95% CI 36.39-45.65); placebo 41.12 ± 14.99 min (95% CI 36.32-45.92); *p* = 0.22) There were no significant adverse events during the study. One patient underwent re-operation after 6h follow up postoperative bleeding due to surgery in the dipyrone group, however this single bleeding case did not have any statistical significance between groups. All randomized patients were taken into statistical analysis.

## DISCUSSION

In this prospective, randomized, double blind, placebo controlled study both intravenous paracetamol and dipyrone yielded similar efficacy with lower cumulative doses of opioid requirement in the first 6 hours during post-tonsillectomy analgesia in pediatric age group when compared with placebo.

Despite it is widely perceived that tonsillectomy needs prompt postoperative analgesic management as it is associated with severe pain, there is lack of a universal approach for these patients which is accepted as ideal^20^^,^^21^. There is no single agent proved to be effective solely, and the management of this clinical dilemma in the contemporary practice mostly comprises of a multimodal approach including regular non-opioid analgesics followed by individually titrated opioid drugs when necessary^21^. There are only a few options for intravenous non-opioid analgesia in postoperative pain management at pediatric age group^22^.

Paracetamol^1^^,^^2^^,^^15^, ^16^, ^17^, ^18^ and dipyrone^1^^,^^5^^,^^6^ are two commonly used intravenous analgesics for this indication. Intravenous paracetamol *vs*. intravenous dipyrone is compared in postoperative pain management in a variety of settings including retinal, maxillofacial, urological, gynecological, and orthopedic, and breast surgery in adult population^23^, ^24^, ^25^, ^26^. Results of these studies revealed similar clinical efficacy between these two agents except one study^25^ implying a potential advantage of paracetamol over dipyrone. In the pediatric age group there is general lack of information on use of medications compared with adults^27^ and there is a strong initiative across Europe to overcome this paucity of data^28^. Our study revealed similar analgesic effect of intravenous paracetamol *vs.* dipyrone reflected by equivalent CHEOPS scores and cumulative pethidine requirement among two active treatment groups.

Nevertheless, paracetamol may be offering some advantage over dipyrone in terms of pain relief score postoperatively. One of the main findings of our study is the lack of difference with regard to CHEOPS scores between active treatment groups and placebo. Previous studies evaluating post tonsillectomy analgesia in pediatric age group evaluating intravenous paracetamol *vs.* opioid drugs^2^^,^^15^^,^^18^ revealed either equivalent or a slightly lower analgesic effect of this drug than pethidine or tramadol; however these studies were not placebo controlled therefore any comment on the analgesic efficacy of intravenous paracetamol compared with placebo cannot be ascertained from them. Our placebo controlled study differs from them particularly by giving an opportunity to comment on effects of these two active treatment regimens when compared with placebo. Although paracetamol at 0.5, 4, 6h and dipyrone at 6h follow up offered some benefit in terms of higher pain relief scores when compared with placebo, it can only be regarded as a minimal clinical benefit over placebo as the pain intensity score was similar between all three groups.

Contrary to the data published by Landwehr et al.^23^, showing a clear benefit by both paracetamol and dipyrone over placebo during adult retinal surgery; another recent placebo controlled study published by Ohnesorge et al.^25^, did not show a significantly different effect on pain intensity of neither intravenous paracetamol nor intravenous dipyrone over placebo in adult patients undergoing breast surgery. Our study revealed a lower cumulative dose of opioid requirement at 6h follow up. Although the cumulative opioid requirement was similar between groups in the study of Ohnesorge et al.^25^, the number of patients requiring opioid analgesia in the first postoperative day was lower in the paracetamol group which can be regarded as a slight opioid sparing effect. On the other hand, while there was no opioid sparing effect of dipyrone in that study, our study also revealed an opioid sparing effect of intravenous dipyrone at 6h follow up over placebo.

The main differences between two studies were the adult *vs*. pediatric age group of the patients recruited and the nature of surgical procedures. Cumulative dose of pethidine requirement were similar in active treatment and placebo groups until 4^th^ hour postoperatively; and the difference between active treatment groups and placebo group was detectable only on the 6^th^ postoperative hour. That finding probably reflects the analgesic effect of fentanyl used intraoperatively during anesthesia induction which may last approximately 4 hours in this age group^29^. Postoperative nausea and vomiting (PONV) was previously correlated with opioid drugs^30^ and pain^31^. In our study, PONV was equivalent among all three groups. Although it was previously shown that increasing opioid dose is associated with increasing PONV incidence^32^^,^^33^ premedication with steroids might have prevented PONV in the placebo group who received a higher cumulative opioid dose. Another factor for this equivalence may be the equivalent pain intensity among all three groups. Duration of stay in post anesthesia care unit was similar in all three groups probably reflecting the equivalent level of sedation and pain intensity among them.

Limitation of our study is the measurement of pain only at rest but not during swallowing, this would yield more information about the clinical efficacy of these agents.

## CONCLUSION

In conclusion, intravenous paracetamol and dipyrone used for analgesia in the early postoperative period after day case tonsillectomy in pediatric age group yielded efficient analgesia with lower cumulative doses of pethidine requirement when compared with placebo. Clinical benefit created by both paracetamol and dipyrone was confined to increased pain relief in 6h follow up and lower cumulative dose of pethidine requirement. Lower pethidine requirement did not lead to an effect on the incidence of opioid-related side effect incidence among groups (PONV, sedation). Contemporary methods used for relieving post tonsillectomy pain in the pediatric age group is still far from ideal and research is still demanded to fulfill this requirement.
